# Seven-year-old Girl with Vomiting, Diarrhea, and Decreased Oral Intake

**DOI:** 10.5811/cpcem.64687

**Published:** 2026-08-05

**Authors:** Mary Girgis, Kathleen Stephanos, Laura J. Bontempo, T. Andrew Windsor

**Affiliations:** * University of Maryland Medical Center, Department of Emergency Medicine and Pediatrics, Baltimore, Maryland; † University of Maryland School of Medicine, Department of Emergency Medicine, Baltimore, Maryland

**Keywords:** thyrotoxicosis, Graves disease, CPC

## Abstract

A seven-year-old girl presented to the pediatric emergency department with three days of nonbloody, nonbilious vomiting, nonbloody diarrhea, and decreased oral intake. She also had weight loss and fatigue over the preceding month. The examination showed persistent tachycardia despite antipyretics and fluids. This presentation addresses the many causes of persistent tachycardia in a child with a diagnostic test sent from the emergency department revealing the ultimate diagnosis.

## CASE PRESENTATION (DR. MARY GIRGIS)

A seven-year-old girl presented with her mother to the pediatric emergency department (ED) for a chief complaint of vomiting and diarrhea for the prior three days. Given the patient’s age, her mother helped provide the history. The mother noted that the patient had been having nonbloody, nonbilious vomiting for the prior three days, coupled with diarrhea without blood or mucous. The patient had been complaining of diffuse abdominal pain during this time. Her mother also noted that she had not been eating very well for the prior few days but did confirm that she had normal urine output. The patient had been feeling fatigued and had been having occasional headaches as well. Her mother noted that she thought her daughter had been losing weight over the preceding month. The patient stated that she felt “like my heart is beating out of my chest.” There were no episodes of fever and no upper respiratory symptoms such as cough, rhinorrhea, or sore throat.

The patient had no medical or surgical history, no known allergies, and took no daily medications. She was homeschooled and the family had not traveled recently. The patient’s two sisters were also sick at home with similar symptoms of vomiting, diarrhea, and abdominal pain. Her parents and siblings were generally healthy. The only known significant family history was a paternal aunt with a history of thyroid disease. The patient was up to date on her vaccinations.

The patient was appropriately alert and oriented for her age. The vital signs on arrival were as follows: temperature, 36.9° Celsius; heart rate, 140 beats per minute (bpm); blood pressure, 106/63 mm Hg; respiratory rate, 22 breaths per minute; and oxygen saturation, 98% on room air. The patient’s body mass index (BMI) was 13.8 kg/m^2^ (less than the fifth percentile). She appeared thin and pale but was interactive with the examination. Her head was normocephalic and atraumatic. Otoscopic examination revealed unremarkable external auditory canals and tympanic membranes bilaterally. The nasal mucosa was unremarkable, without congestion or rhinorrhea. Mucous membranes were moist, and the oropharynx was clear without exudate or posterior oropharyngeal erythema. There was no ocular discharge, and conjunctivae were normal bilaterally.

The patient had nontender bilateral anterior cervical lymphadenopathy. Her heart had a regular tachycardic rhythm. Pulses were normal, and heart sounds were without any murmurs, rubs, or gallops. She had a normal respiratory effort and clear lung sounds without any wheezing, stridor or decreased breath sounds. Her abdomen was soft and flat without any distension. She was mildly tender diffusely but without guarding or rebound, and no masses or hernias were noted. There was mild hepatomegaly without splenomegaly. Her bowel sounds were normal. Her musculoskeletal, skin, and neurological exams were all normal, and genitourinary exam was deferred.

**Image 1:**
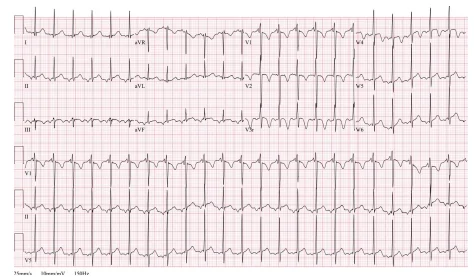
Electrocardiogram of a seven-year-old girl with vomiting, diarrhea, and decreased oral intake.

**Image 2:**
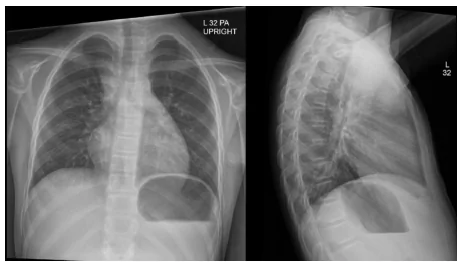
Chest radiograph of a seven-year-old girl with vomiting, diarrhea. and decreased oral intake.

The patient’s initial laboratory studies are shown in the table. An electrocardiogram (ECG) (Image 1) and chest radiograph (Image 2) were also obtained.

On reassessment, the patient was noted to have continued tachycardia with a heart rate of 150 bpm and was febrile to 38.1° Celsius. She was given a 20 mL/kg intravenous fluid bolus and oral antipyretics. She was reassessed approximately one hour later; despite volume expansion, her heart rate was unchanged, but her fever had improved. Two hours later, a new pediatric ED team took over her care. At that point, additional testing was ordered, and a diagnosis was made.

## CASE DISCUSSION (DR. KATHLEEN STEPHANOS)

When I first began to review this case, I was struck by how vague the initial symptoms were in this child. Vomiting was one of the complaints, but this is so prevalent in young children that it was even removed from the algorithm for pediatric head trauma.^1^ The combination of fatigue, headache, and gastrointestinal symptoms may point many clinicians toward a diagnosis of viral illness. This is one of the most common diagnoses in the pediatric ED; however, these symptoms can also be the presenting complaint for more serious pathology, as is likely the case in this patient.^2^ The frequency of these symptoms can result in confirmation bias, recency bias, and anchoring bias. The fact that the patient’s siblings had similar symptoms further pushes the clinician toward premature closure. However, anchoring and bias must be avoided to ensure that a correct diagnosis is made.

To recap, this is a case of a homeschooled 7-year-old who presented to an ED with vomiting, diarrhea, fatigue, headaches, palpitations, and weight loss. Her examination was notable for tachycardia and diffuse abdominal discomfort but was otherwise reassuring. She was later noted to have a fever. Her chest radiograph showed clear lung fields with no pulmonary edema and a normal cardiac silhouette. Her ECG was notable for an ectopic atrial tachycardia, as identified by the fast rate with an upright P wave in lead aVR. The laboratory tests, which I will discuss in more detail, showed mild leukopenia, anemia, hyponatremia, mild transaminitis, and an elevated N-terminal pro-brain natriuretic peptide (NT-proBNP).

From the patient’s history I learned several things about the child that stand out and begin to help me formulate a differential beyond the simple “viral illness.” When hearing that the patient attended home school, it is worth noting that home-schooled children have higher rates of nonvaccination or undervaccination; thus, this may increase the risk of vaccine-preventable diseases, particularly if vaccination status is unknown or unreported.^3^ While important to consider, ultimately this child was (or at least was reported to be) fully vaccinated, and none of the patient’s symptoms fit well with any of the common illnesses that would be covered by the typical vaccine schedule.

I next began to approach the topic of the child’s weight loss. In children, weight loss is almost always a red flag for serious pathology, often due to indolent processes, and warrants immediate investigation.^4^ Because children are expected to grow continuously, true weight loss is particularly concerning. When evaluating a child with weight loss or poor weight gain, it is essential to confirm this using a growth chart. Typically, failure to thrive first appears as poor weight gain, reflected by declining weight percentiles, before actual weight loss occurs. Later findings include decreased height growth velocity followed by small-for-age head circumference. Notably patterns that show short stature or small head circumference before inappropriate weight gain should prompt other investigation into nonnutritional causes, further supporting the importance of growth chart use. In this patient, I was not given these other variables. Inadequate intake can cause weight loss in cases of socioeconomic instability or disordered eating. Neither of these are clearly indicated by the history of this patient. Caloric losses could be from inflammatory bowel disease, celiac disease, pancreatic insufficiency, or protein-losing enteropathies. The history of diarrhea may support this; however, it appears that the patient’s weight loss preceded any gastrointestinal symptoms. Increased metabolic demand can also cause weight loss in the cases of congenital heart disease, chronic kidney disease, severe anemia, malignancies, hyperthyroidism, or diabetes.

With that differential in mind, I began to focus on the abdominal pain, vomiting, and diarrhea with vague tenderness on exam. This did initially expand my differential slightly to include a focal intraabdominal process such as cholecystitis or pancreatitis, vasculitis, and myocarditis; however, none of these fit the patient’s clinical picture. Pediatric patients without a history of hereditary hemoglobinopathy typically do not have cholecystitis, while children without known cystic fibrosis rarely have pancreatic issues unless they are anatomic in nature or idiopathic. While we do not know whether this child receives regular medical care, it would be unusual for a child to reach the age of seven years without identifying these diagnoses. Importantly, the examination did not demonstrate a focal area of tenderness to support specific organ involvement of her symptoms. Vasculitis, on the other hand, may present with vague gastrointestinal symptoms, which may be present before rash development. However, this only occurs in 10–40% of patients, and the rash is typically the hallmark of diagnosis.^5^

I reviewed the ECG, which showed an ectopic atrial tachycardia. This is atypical for children and usually indicates a structural cardiac abnormality (such as an atrial mass), myocarditis, or heart failure. Rarely, it can also occur in children with excessive cardiac output due to excessive metabolic demand. Overall, this is an unusual rhythm and made me more concerned that something more is going on than a simple virus. Notably, the ECG itself does not have signs of hypertrophy. The downward T wave, predominant S wave, and lack of dominant R in V1 indicate no evidence of right ventricular hypertrophy, while the R wave in V6 not reaching the baseline of V5 roughly estimates no evidence of left ventricular hypertrophy.^6^

The laboratory values allowed us to remove some of the diagnoses from the differential for this patient. The blood glucose was not elevated, removing diabetes as a possibility. The patient did have anemia, although not to the level that would explain her tachycardia or require emergent intervention. Because there is no history to suggest acute blood loss, the anemia could be secondary to an underlying condition and is unlikely to be the primary cause of all the patient’s presenting symptoms. The mild elevation of liver enzymes and mild hyponatremia may be associated with many diagnoses and are more suggestive of nonspecific inflammation than a primary diagnosis. The NT-proBNP level is elevated. This is generally elevated due to a cardiac cause of symptoms, such as heart failure or myocarditis, both of which could occur because of a recent viral infection, as described in this patient. Infants typically will present with vague symptoms for either of these diagnoses, with tachypnea or sweating during feeds, or poor feeding in general, while older children may have more typical symptoms as seen in adults with poor exercise tolerance and possible fluid overload.^7^

Elevated NT-proBNP may also be due to high output states, and this is a common cause of elevation in children. Importantly, in this case the chest radiograph did not demonstrate cardiac enlargement or fluid overload. While myocarditis remains a concern, typically the presentation for myocarditis is not this indolent.

Putting all the information together, I kept coming back to the initial weight loss coupled with her persistent tachycardia. As the case went on, the patient continued to be tachycardic. She did have a fever, but even with defervescence, the tachycardia persisted. Her weight loss resulting in a BMI below the fifth percentile means that the process she is dealing with likely was present for a long time. The weight loss was possibly worsened by the diarrhea and possible viral infection that she and her sibling shared, rather than being caused by the viral illness itself. Given the reassuring laboratory values and no evidence of supraventricular tachycardia on her electrocardiogram, this eliminates many common causes of pediatric tachycardia. It seems, from the ECG and elevated NT-proBNP with a normal chest radiograph that while there is a cardiac impact of the underlying disease, it does not appear to be the primary cause.

Notably the patient’s blood pressure remains normal despite fluid boluses, again pointing away from a cardiogenic shock picture. Coupling the findings of severe weight loss with persistent tachycardia and ultimately a fever, I am suspecting a potential underlying hypermetabolic disorder, particularly hyperthyroidism. Most cases of hyperthyroidism in children are due to the autoimmune disease known as Graves.^8^ Other causes of hypermetabolism in children include inborn errors of metabolism, severe combined immunodeficiency, oncologic processes, or burns. Inborn errors of metabolism and immunodeficiency do not usually present in an otherwise healthy 7-year-old with no prior history. An oncologic process could be considered but she has no other symptoms of night sweats, lymphadenopathy, or splenomegaly to suggest this etiology. Although she did have mild hematopoietic suppression, it was more consistent with her recent viral illness than the profound suppression seen in pediatric oncologic disorders. Clearly history and physical examination eliminate burns. Ultimately, the additional labs I want to obtain include a thyroid stimulating hormone (TSH) level in conjunction with a thyroxine (T4) level to assess for hyperthyroidism.

## CASE OUTCOME (DR. MARY GIRGIS)

The diagnostic test was a thyroid panel which showed a TSH less than 0.01 mIU/L (reference range, 0.5–4.50 mIU/L) and an elevated free T4 level of 6.2 ng/dL (0.6–2.5 ng/dL), demonstrating that the patient was suffering from thyrotoxicosis. The patient was started on propranolol (8 mg) three times a day and methimazole (20 mg daily) and was admitted to the pediatric floor. While admitted, the patient was positive for thyroid-stimulating immunoglobulin and TSH receptor antibodies (TRAb) which confirmed that she had Graves disease.

## RESIDENT DISCUSSION (DR. MARY GIRGIS)

Thyroid disease occurs on a spectrum. The starting point is hyperthyroidism, a biochemical definition in which the thyroid produces excess triiodothyronine (T3) and T4, causing a negative feedback loop to the pituitary gland, which results in a low TSH. Thyrotoxicosis occurs when a patient has clinical symptoms because of these abnormal values. Thyrotoxicosis is relatively common in adults, but it is rare in preadolescent children. Graves disease accounts for approximately 95% of cases of hyperthyroidism in children, with an incidence of only about 1 to 3 per 100,000 children per year before 15 years of age.^8^ The term “thyroid storm” describes the development of end-organ dysfunction secondary to thyrotoxicosis.^9^ This condition is an endocrine emergency. The incidence of thyroid storm in patients younger than 15 years is estimated at 0.9 per 100,000 population per year, according to a United Kingdom study.^10^ For all ages in the United States the incidence of thyroid storm ranges between 0.2 and 0.76 per 100,000 persons per year, with an incidence of 4.8–5.6 per 100,000 hospitalized patients.^9^ The mortality of thyroid storm was historically quite high, but newer data suggest that it is approximately 1.2%–3.6% in the United States.^9^

Usually, thyroid storm can be linked to a precipitating event, such as infection, surgery, or medication noncompliance.^11^ It can also be from an autoimmune process such as Graves disease, as in this case. Graves disease occurs when TRAb bind to the thyrotropin receptor, causing overproduction of thyroid hormone and thyroid growth. This can continue unchecked, which results in many downstream effects. These effects present as hyperthyroidism, which can range from mild to severe and can lead to thyrotoxicosis if untreated.^12^

The clinical presentation of thyrotoxicosis is varied; thyrotoxicosis can be mild to severe, and it can affect most organ systems. For example, patients can have cardiovascular effects that range from tachycardia and atrial fibrillation to decompensated heart failure. Patients may have neurological symptoms such as agitation, anxiety, delirium, psychosis, and even coma. Patients can present with gastrointestinal symptoms such as nausea, vomiting, or diarrhea. In severe cases, patients may have hepatic failure. Patients may also have systemic signs such as hyperpyrexia and sweating. On exam, a patient may have tremors, and they may be warm and sweaty. They may have a lid lag, exophthalmos, and a goiter. Other laboratory abnormalities that can be seen include hypercalcemia, hyperglycemia, elevated liver enzymes, leukocytosis, or leukopenia.^11^

The treatment of thyrotoxicosis and thyroid storm involves a combination of medications that both inhibit the synthesis of thyroid hormone and block the conversion of T4 into T3. Patients should first be treated with a beta blocker for symptomatic management of sympathetic hyperactivity. Several beta blocker options exist for clinical management. Propranolol is frequently used because it partially blocks conversion of throxine (T4) to tridothyrodine (T3); however, atenolol and metoprolol are more cardio selective. Notably, atenolol should be avoided in pregnant patients.^13^ The dose for propranolol is 10–40 mg three to four times a day in adults and one to four mg/kg/day in divided doses for pediatric patients, titrating based on the patient’s heart rate and blood pressure.^14^ After controlling the sympathetic hyperactivity, patients should then be given an antithyroid drug such as methimazole or propylthuracil (PTU). These block the synthesis of thyroid hormone.^12^, ^13^ Because methimazole, is teratogenic, PTU is preferred in pregnancy. Patients may also receive iodine, which inhibits the release of stored thyroid hormone. This must be given after the sympathetic pathway has been blocked by administration of methimazole or PTU. Steroid administration is another option because it decreases peripheral conversion of T4 to T3. It can be helpful if there are concerns for concomitant adrenal insufficiency.^13^, ^14^

Thyrotoxic patients presenting to the ED should be admitted and monitored for improvement of symptoms. Patients in thyroid storm may need to be admitted to an intensive care unit. They should be followed outpatient by an endocrinologist because medication titration is often necessary.

## FINAL DIAGNOSIS

Thyrotoxicosis in a pediatric patient secondary to Graves disease

**KEY TEACHING POINTS**

- Weight loss in children is abnormal and often portends serious illness.
- Thyrotoxicosis and thyroid storm can affect any organ system and can lead to end-organ dysfunction.
- Consider thyroid storm in patients who have refractory hyperpyrexia and/or tachycardia that is unexplained by other processes.
- Remember that methimazole and atenolol are generally avoided in pregnancy.

## Figures and Tables

**Table:** Laboratory values of a seven-year-old girl who presented with vomiting, diarrhea. and decreased oral intake.

Test	Patient value	Reference Range
Complete Blood Count		
White Blood Cell	3.8 K/mcL	4.5 – 11 K/mcL
Hemoglobin	9.5 g/dL	11.9 – 15.7 g/dL
Hematocrit	29.8%	35.0 – 45.0%
Platelets	228 K/mcL	153 – 367 K/mcL
Complete Metabolic Panel		
Sodium	130 mmol/L	136 – 145 mmol/L
Potassium	4.1 mmol/L	3.5 – 5.1 mmol/L
Chloride	99 mmol/L	98 – 107 mmol/L
Bicarbonate	25 mmol/L	21 –30 mmol/L
Anion Gap	7	5–16
Blood urea nitrogen	10 mg/dL	7 – 17 mg/dL
Creatinine	0.23 mg/dL	0.52 – 1.04 mg/dL
Glucose	88 mg/dL	70–100 mg/dL
Calcium	9.3 mg/dL	8.8–10.8 mg/dL
Total Protein	6.2 g/dL	6.2–8.1 g/dL
Albumin	3.6 g/dL	3.2 – 4.6 g/dL
Total bilirubin	0.6 mg/dL	0.3 – 1.2 mg/dL
Direct bilirubin	0.2 mg/dL	≤0.4 mg/dL
Aspartate aminotransferase	71 units/L	14 – 36 units/L
Alanine aminotransferase	46 units/L	0 – 34 units/L
Alkaline phosphatase	193 units/L	38 – 126 units/L
Additional Labs		
Troponin I	<0.02 ng/mL	≤0.06 ng/mL
N-terminal pro-brain natriuretic peptide	484.0 pg/mL	20.0 – 217.0 pg/mL

*K*, thousands; *mcL*, microliter; *g*, grams; *dL*, deciliter; *mmol*, millimole; *L*, liter; *mg*, milligram; *mcg*, microgram; *mL*, milliliter; *ng*, nanogram; *pg*, picogram.

## References

[1] Borland ML, Dalziel SR, Phillips N (2018). Vomiting with head trauma and risk of traumatic brain injury. Pediatrics.

[2] McDermott KW, Stocks C, Freeman WJ (2018). Overview of pediatric emergency department visits, 2015. Healthcare Cost and Utilization Project (HCUP) Statistical Briefs [Internet].

[3] Mohanty S, Joyce CM, Delamater PL (2020). Homeschooling parents in California: attitudes, beliefs and behaviors associated with child’s vaccination status. Vaccine.

[4] Levinson B (2025). Evaluation of weight loss in children beyond 6 months. AAP Pediatric Care Online.

[5] Roache-Robinson P, Killeen RB, Hotwagner DT (2024). IgA vasculitis (Henoch-Schönlein purpura). StatPearls.

[6] Evans W, Acherman R, Mayman G (2010). Simplified pediatric electrocardiogram interpretation. Clin Pediatr.

[7] Jayaprasad N (2016). Heart failure in children. Heart Views.

[8] Hanley P, Lord K, Bauer AJ (2016). Thyroid disorders in children and adolescents: a review. JAMA Pediatr.

[9] Farooqi S, Raj S, Koyfman A, Long B (2023). High risk and low prevalence diseases: thyroid storm. Am J Emerg Med.

[10] Williamson S, Greene SA (2010). Incidence of thyrotoxicosis in childhood: a national population-based study in the UK and Ireland. Clin Endocrinol (Oxf).

[11] Sarlis NJ, Gourgiotis L (2003). Thyroid emergencies. Rev Endocr Metab Disord.

[12] American Thyroid Association (2023). Graves’ disease.

[13] Ross DS, Burch HB, Cooper DS (2016). 2016 American Thyroid Association guidelines for diagnosis and management of hyperthyroidism and other causes of thyrotoxicosis. Thyroid.

[14] Shenoi RP, Timm N, Committee on Drugs; Committee on Pediatric Emergency Medicine (2020). Drugs used to treat pediatric Eemergencies. Pediatrics.

